# Timing of transcription during the cell cycle: Protein complexes binding to E2F, E2F/CLE, CDE/CHR, or CHR promoter elements define early and late cell cycle gene expression

**DOI:** 10.18632/oncotarget.10888

**Published:** 2016-07-28

**Authors:** Gerd A. Müller, Konstanze Stangner, Thomas Schmitt, Axel Wintsche, Kurt Engeland

**Affiliations:** ^1^ Molecular Oncology, Medical School, University of Leipzig, 04103 Leipzig, Germany; ^2^ Computational EvoDevo Group, Department of Computer Science and Interdisciplinary Center for Bioinformatics, University of Leipzig, 04107 Leipzig, Germany

**Keywords:** transcriptional repression, E2F, CHR transcriptional element, DREAM complex, p53 tumor suppressor

## Abstract

A central question in cell cycle control is how differential gene expression is regulated. Timing of expression is important for correct progression through the cell cycle. E2F, CDE, and CHR promoter sites have been linked to transcriptional repression in resting cells and activation during the cell cycle. Further, the DREAM complex binds CHR or CDE/CHR elements of G_2_/M genes resulting in repression during G_0_/G_1_. Here, we show that DREAM also binds to E2F sites of S phase genes in quiescence and upon p53 activation. Furthermore, we describe a novel class of promoter sites, the CHR-like elements (CLE), which can support binding of DREAM to E2F elements. Activation of such S phase genes is achieved through binding of E2F1-3/DP complexes to E2F sites. In contrast, the activating MuvB complexes MMB and FOXM1-MuvB bind to CHR elements and mediate peak expression in G_2_/M. In conclusion, data presented here in combination with earlier results leads us to propose a model that explains how DREAM can repress early cell cycle genes through E2F or E2F/CLE sites and late genes through CHR or CDE/CHR elements. Also p53-dependent indirect transcriptional repression through the p53-p21-Cyclin/CDK-DREAM-E2F/CLE/CDE/CHR pathway requires DREAM binding to E2F or E2F/CLE sites in early cell cycle genes and binding of DREAM to CHR or CDE/CHR elements of late cell cycle genes. Specific timing of activation is achieved through binding of E2F1-3/DP to E2F sites and MMB or FOXM1-MuvB complexes to CHR elements.

## INTRODUCTION

Cell cycle-dependent transcriptional regulation is a hallmark of the cell division cycle [1]. MuvB complexes play a central role in the coordination of periodic gene expression by repressing early and late cell cycle genes in G_0_/G_1_ and by activating late gene expression during S, G_2_ and mitosis [2]. The DREAM complex consists of p130 /p107, E2F4/5, DP1 and the MuvB core components LIN9, LIN37, LIN52, LIN54, and RBBP4 [3, 4]. We have recently shown that DREAM binds to CHR elements or CDE/CHR tandem sites of late cell cycle gene promoters in non-proliferating cells or upon activation of p53 [5–10]. In these cases, the complex predominantly binds to CHR elements. This was shown by mutation of CHR sites, which led to a complete loss of DREAM binding despite presence of CDE sites. In contrast, mutation of CDE sites leads only to a reduction of DREAM binding and repression [9]. The binding behavior of the complex to single CHR and tandem CDE/CHR sites can be explained by the structure of the complex containing two components that interact with different DNA binding sites. LIN54 has been shown to bind to CHR sites *in vitro* [11], and E2F4/DP1 interact with E2F binding sites [12, 13]. CDE sites are related to E2F binding sites but do not resemble canonical E2F elements [14]. More importantly, many CHR elements neither require a CDE nor an E2F site for transcriptional regulation and DREAM binding [9]. Thus, CHR sites can function alone, independently of E2F or CDE sites.

The CHR is necessary not only for repression in G_0_/G_1_ but also for activation in late cell cycle phases. This further underlines the significance of the CHR as the central element in cell cycle-dependent regulation of late cell cycle genes [8, 9]. During progression through the cell cycle, p130 is hyperphosphorylated by cyclin/cdk complexes, which results in dissociation of the DREAM components p130, E2F4, and DP1 from the MuvB core [15]. In S phase, the MuvB components interact with B-MYB. The B-MYB-MuvB (MMB) complex then recruits FOXM1 and B-MYB is degraded. The FOXM1-MuvB complex stimulates maximum expression of late cell cycle genes in G_2_/M [2, 3, 16]. Both complexes bind to their target genes through CHR sites while CDE or E2F sites are not involved [8, 9, 17].

Having analyzed binding of DREAM, MMB, and FOXM1-MuvB to CHR and CDE sites of late cell cycle genes previously, we aimed for a detailed analysis of DREAM binding and cell cycle-dependent regulation of genes expressed with a maximum in S phase. It has long been recognized that cell cycle-dependent transcription of such genes is regulated through E2F binding sites via activating (E2F1-3) and repressing (E2F4-5) E2F factors together with the pocket proteins pRB, p130, and p107 [18, 19]. p130 and p107 mainly interact with E2F4 and E2F5, while pRB preferentially binds E2F1-3 [20, 21]. Furthermore, because of a specific phosphate-binding pocket that has been identified in p130/p107 which binds to phosphorylated LIN52 of the MuvB core, only p130 or p107 but not pRB are part of the DREAM complex [15]. However, the specific functions of the pocket proteins in regulating early cell cycle genes during proliferation, quiescence, senescence, and differentiation are still not completely understood. While p130 and p107 seem to share largely overlapping functions and regulate the same set of genes, pRB can influence expression of different targets depending on the cell type and the cellular context [22, 23].

E2F binding sites were found to be enriched in DREAM target genes that are mainly expressed in S phase [3, 8]. Furthermore, such genes are upregulated in G_0_ upon knockdown or knockout of DREAM components [3, 24]. Based on these findings, we wondered whether DREAM can bind to single E2F sites or whether other elements are necessary to recruit the complex to promoters of S phase genes. Since we have shown that DREAM can interact with CHR and CDE binding sites in G_2_/M promoters, we reasoned that the complex may mainly bind to E2F sites of early cell cycle genes but that binding could be supported via interactions of LIN54 with CHR-like elements (CLE) located 4 bp downstream from E2F sites. To verify this hypothesis, we identified and analyzed candidate genes harboring different potential DREAM binding sites in their core promoters: *TTK* (CDE/CHR); *INCENP* (CHR); *ORC1*, *Atad5*, *Gins1* (E2F); *RAD51*, *CDC45*, *Mybl2* (E2F/CLE). The genes and their promoters were tested for cell cycle-dependent expression and protein binding. Furthermore, DREAM binding to promoters also appears to be central for p53-mediated transcriptional repression [10, 25]. Therefore, we investigated the activity of the promoters upon p53 activation. p53 activates the gene of the cyclin-dependent kinase inhibitor p21/CDKN1A. Increasing cdk inhibitor levels cause hypophosphorylation of p130 which then allows formation of DREAM [26]. This pathway finally yields a switch from activating MMB to repressing DREAM complexes, which both bind DNA through CHR elements [10]. Here, we address the important question whether this mechanism of transcriptional repression by p53 also applies to S phase genes.

Taken together, we provide a comprehensive model for the control of cell cycle- and p53-dependent transcriptional regulation by mammalian MuvB-derived complexes.

## RESULTS

### Identification of potential CHR-like elements, CLEs, in promoters of S phase genes

We hypothesized that DREAM binds to the promoters of S phase genes through E2F binding sites and that additional CHR-like elements (CLE) support recruitment of the complex. To test this hypothesis, we screened DREAM target genes with an expression maximum in S phase containing phylogenetically conserved E2F elements in a region of +/−200 bp around the transcription start sites [8] for CLEs that are located 4 bp downstream of the E2F sites (Supplementary Table S2). These potential coregulatory regions exhibit high sequence variability. Interestingly, only one gene with a canonical CHR (TTTGAA) was detected as an exemption, *KIAA1731/CEP295*. Several genes harbor elements that are related to functional CHR sites but differ in one or two nucleotides from the consensus. These genes include *CDC6* (TGGGAA), *CDC25A* (TAGGAA), *CDC45* (CTTGAC), *MCM3* (TCGGAA), *RAD51* (TCTGAA), and *TCF19* (TGTGAA).

### E2F and E2F/CLE sites control S phase genes while CHR and CDE/CHR sites regulate genes expressed with a maximum in G_2_ phase or mitosis

**Figure 1:**
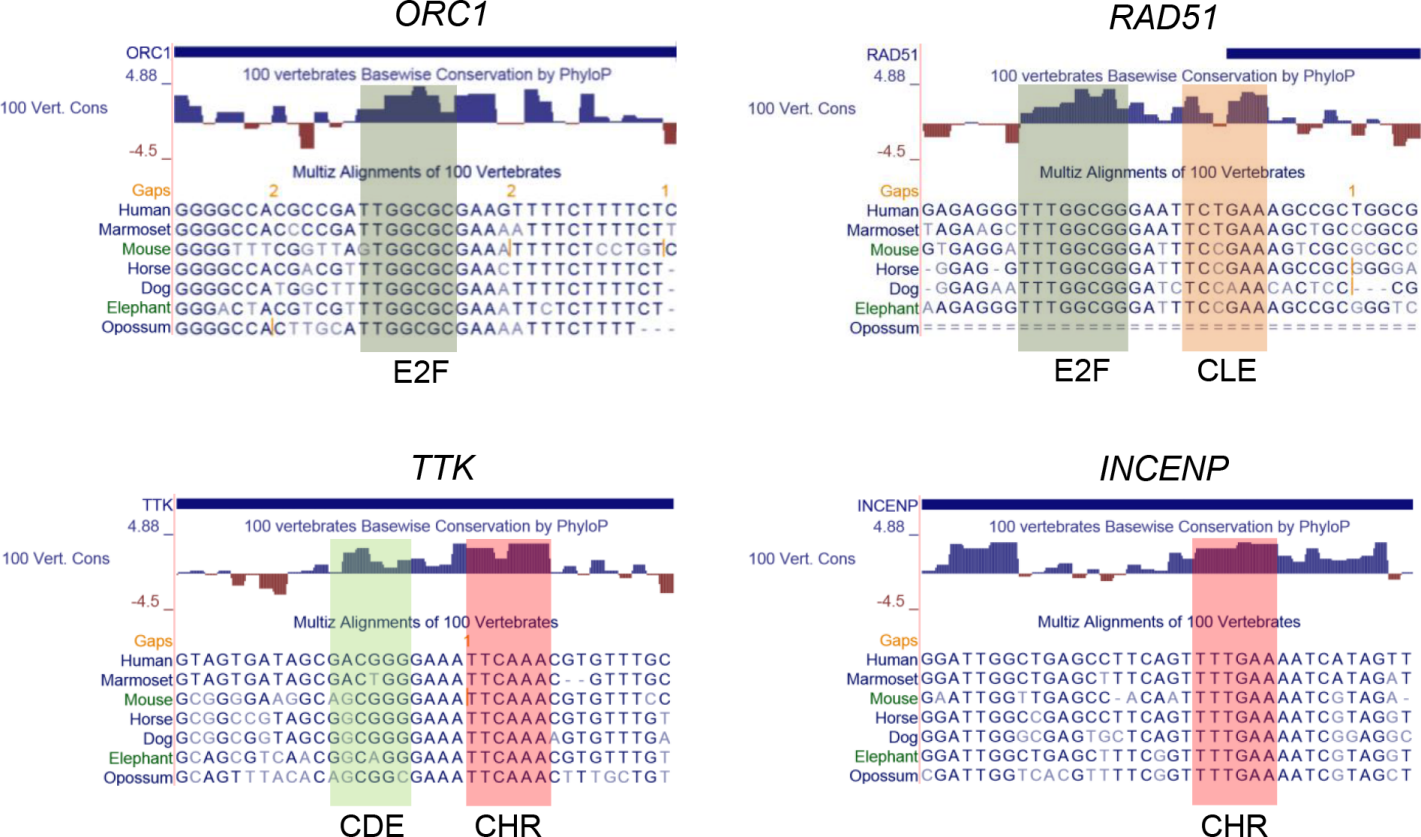
Early and late cell cycle genes exhibit specific evolutionary conserved single and tandem elements in their promoters Potential regulatory elements of *ORC1*, *RAD51*, *TTK*, and *INCENP* were identified with the UCSC genome browser by aligning promoter sequences of seven mammalian species and by applying the 100 vertebrate conservation track [52].

To test whether DREAM can bind to E2F sites and whether downstream CLEs contribute to DREAM-DNA interactions, we selected several candidate genes with putative DREAM binding sites for a detailed analysis. The examples chosen represent the four site combinations E2F, E2F/CLE, CHR, and CDE/CHR. *ORC1* (Origin recognition complex subunit 1) harbors an E2F binding site (Figure 1) that has been shown to be required for cell cycle-dependent transcription [27]. The region starting five nucleotides downstream of the E2F site, TTTTCT, is not related to a CHR. In the *RAD51* promoter an E2F element has also been described [28]. We identified a potential CLE downstream of the E2F site (Figure 1). *MYBL2* (B-MYB) and *CDC45* were tested as candidates for E2F/CLE tandem sites and *Atad5* (ATPase family AAA domain-containing protein 5) as well as *Gins1* as examples for single E2F elements (Supplementary Figure S1). Furthermore, we selected *TTK* and *INCENP* (inner centromere protein) as potential CDE/CHR and CHR genes, respectively, to analyze and compare promoter regulation and protein-DNA interactions with the potential E2F and E2F/CLE sites (Figure 1).

**Figure 2:**
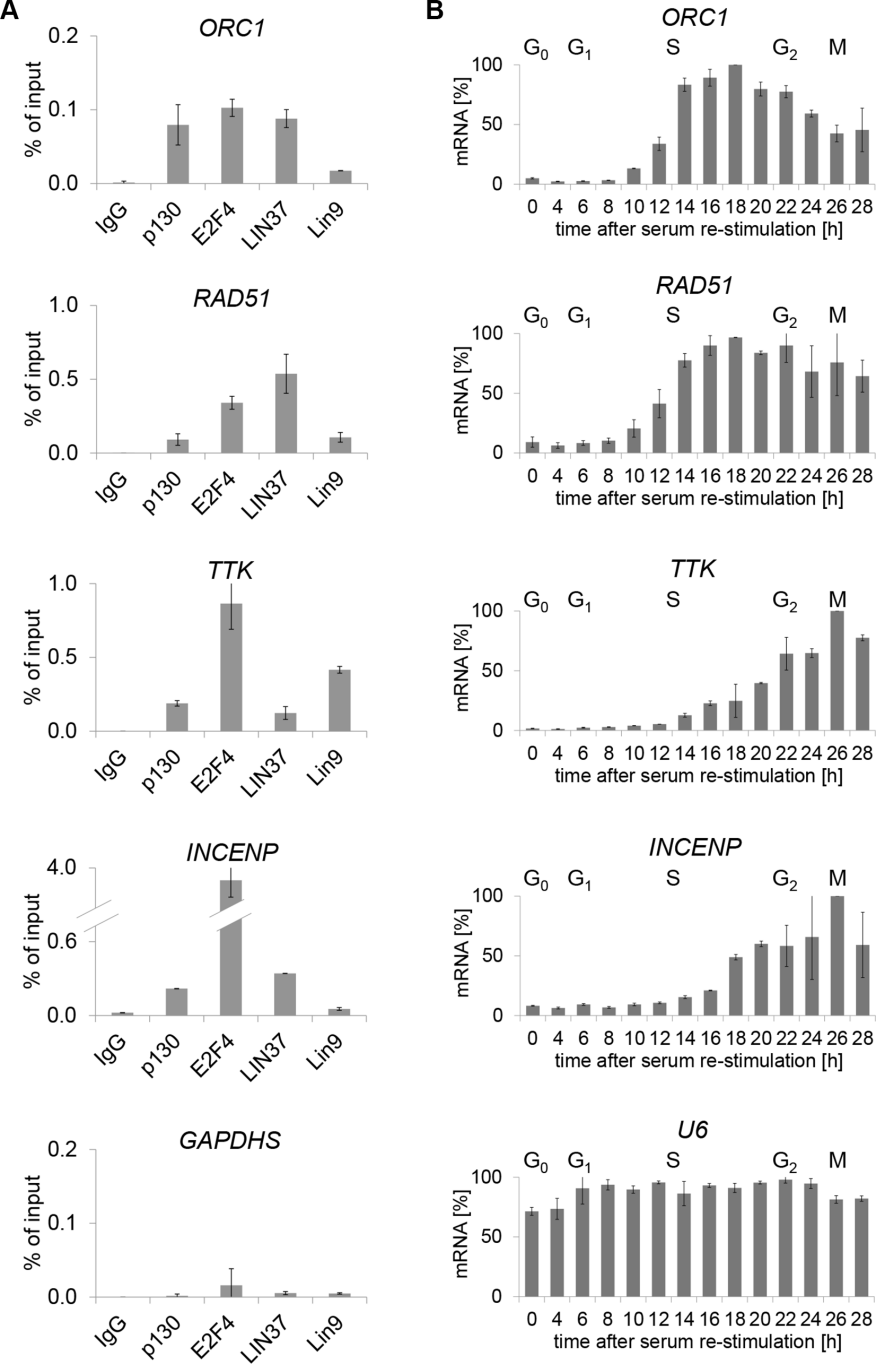
*ORC1* and *RAD51* mRNA is mainly expressed in S phase, while expression of *TTK* and *INCENP* peaks in G_2_/M. All four genes are bound by DREAM in G_0_ (**A**) Binding of the DREAM components p130, E2F4, Lin37, and Lin9 was analyzed via ChIP-qPCR in serum starved T98G cells. A non-targeting antibody (IgG) served as a negative control. (**B**) mRNA expression as measured in serum-starved and re-stimulated HFF cells.

First, we tested whether the genes are indeed DREAM targets and in which phase of the cell cycle their maximum expression is reached. Binding of the DREAM components E2F4, p130, LIN37, and LIN9 was analyzed in serum-starved T98G cells by ChIP-qPCR. DREAM proteins were clearly enriched at all promoters tested (Figure 2A, Supplementary Figure S1). In order to quantify cell cycle-dependent mRNA expression, HFF fibroblasts were arrested in G_0_ by serum starvation followed by serum restimulation. Cells were collected every three hours and analyzed for cell cycle distribution by flow cytometry (Supplementary Figure S1). Semi-quantitative qPCR revealed that the genes containing E2F or E2F/CLE elements were expressed with a maximum in S phase, while expression of *TTK* and *INCENP* as representatives for CDE/CHR and CHR genes, respectively, peaked in G_2_/M phases (Figure 2B, Supplementary Figure S1).

**Figure 3:**
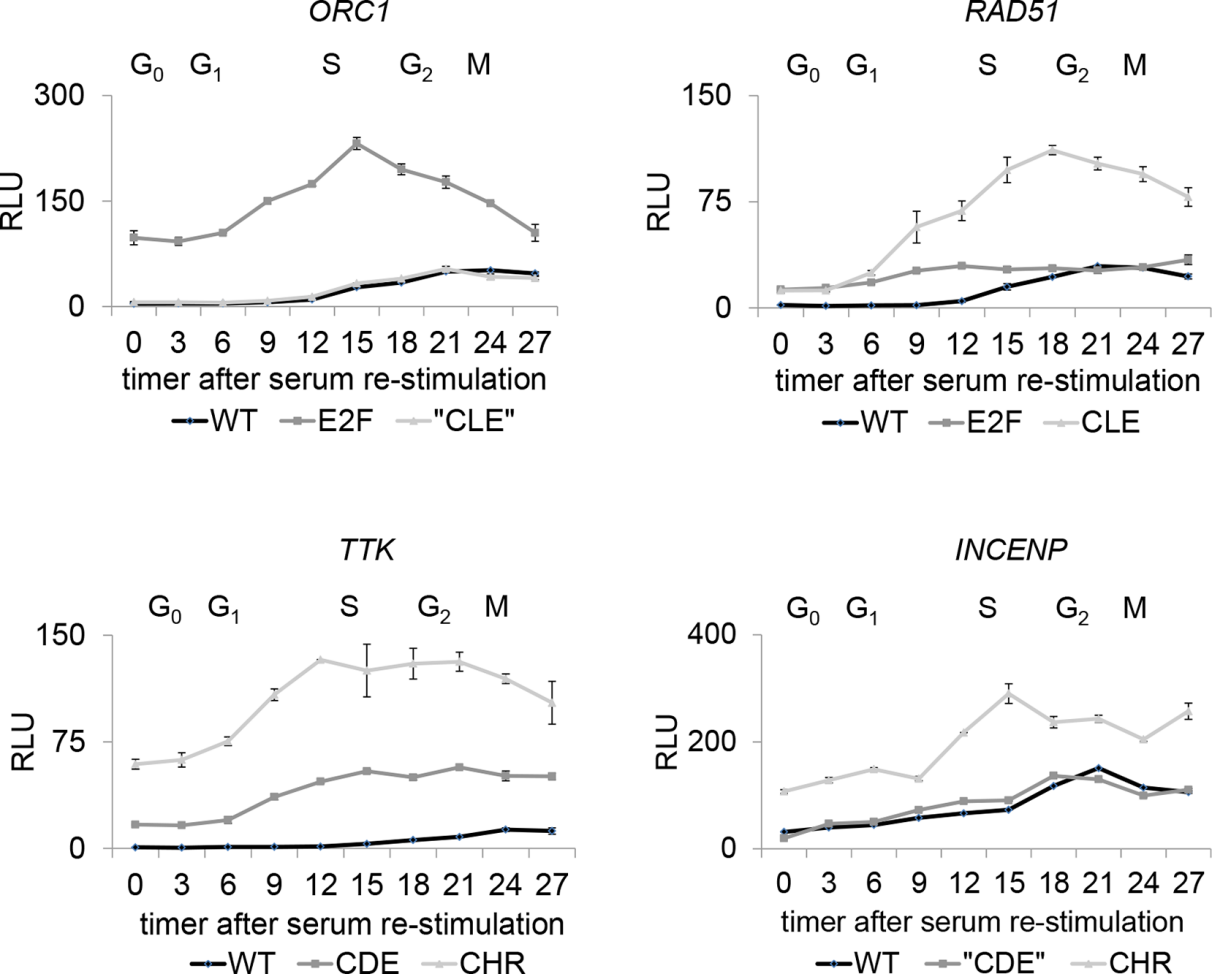
Different DNA elements regulate cell cycle-dependent promoter activity of early or late cell cycle genes NIH3T3 cells were transfected with luciferase reporter constructs of wild-type (wt) and mutant (CDE, CHR, E2F, CLE) promoters and synchronized in G_0_ by serum starvation. “CDE” and “CLE” represent non-functional elements. Cells were stimulated to re-enter the cell cycle be serum addition and collected every three hours. Normalized luciferase relative light units (RLU) are given.

To test whether the identified promoter elements mediate cell cycle-dependent transcriptional regulation, we performed luciferase reporter assays. Promoter acitivities of wild-type and mutant constructs were analyzed in synchronized NIH3T3 cells in different phases of the cell cycle. The activities of all wild-type promoters were low in G_0_ and G_1_ and increased during S phase. Mutation of the E2F sites in *ORC1* and *RAD51* or the CHR in *INCENP* and *TTK* promoters led to a strong derepression in G_0_/G_1_ and a general deregulation in all cell cycle phases (Figure 3). Importantly, *RAD51*, *CDC45*, and *Mybl2* promoters were also deregulated upon mutation of potential CLE sites. This shows that the elements indeed participate in cell cycle-dependent transcriptional regulation (Figure 3, Supplementary Figure S1). Similar observations with moderate deregulation were made for the potential CDE in the *TTK* promoter (Figure 3). In contrast, corresponding mutations in promoters without predicted CLE (*ORC1*) or CDE (*INCENP*) sites did not deregulate the promoters (Figure 3). Taken together, we show that E2F and CHR elements are central promoter elements genes expressed in early and late phases of the cell cycle, respectively. Importantly, CLEs that are located 4 bp downstream of functional E2F sites participate in the cell cycle-dependent regulation of S phase genes (Table 1).

**Table 1:** Properties of promoter elements distinguishing expression of early and late cell cycle genes

	E2F	CLE	CDE	CHR
commonmotifs	TTSSCGC	CTTGAA,CTTGAC,TCTGAA	GGGCGG,TGGCGG	TTTGAA,TTCGAA,TTTAAA
max.expression	S phase	S phase	G_2_/M	G_2_/M
repression	DREAM,E2F/pRB	DREAM [CLE + E2Fsite]	DREAM [CDE + CHRsite]	DREAM
activation	E2F1-3/DP	–	–	MMB,FOXM1-MuvB

### DREAM is recruited to promoters of early and late cell cycle genes through E2F, E2F/CLE, CHR or CDE/CHR sites

**Figure 4:**
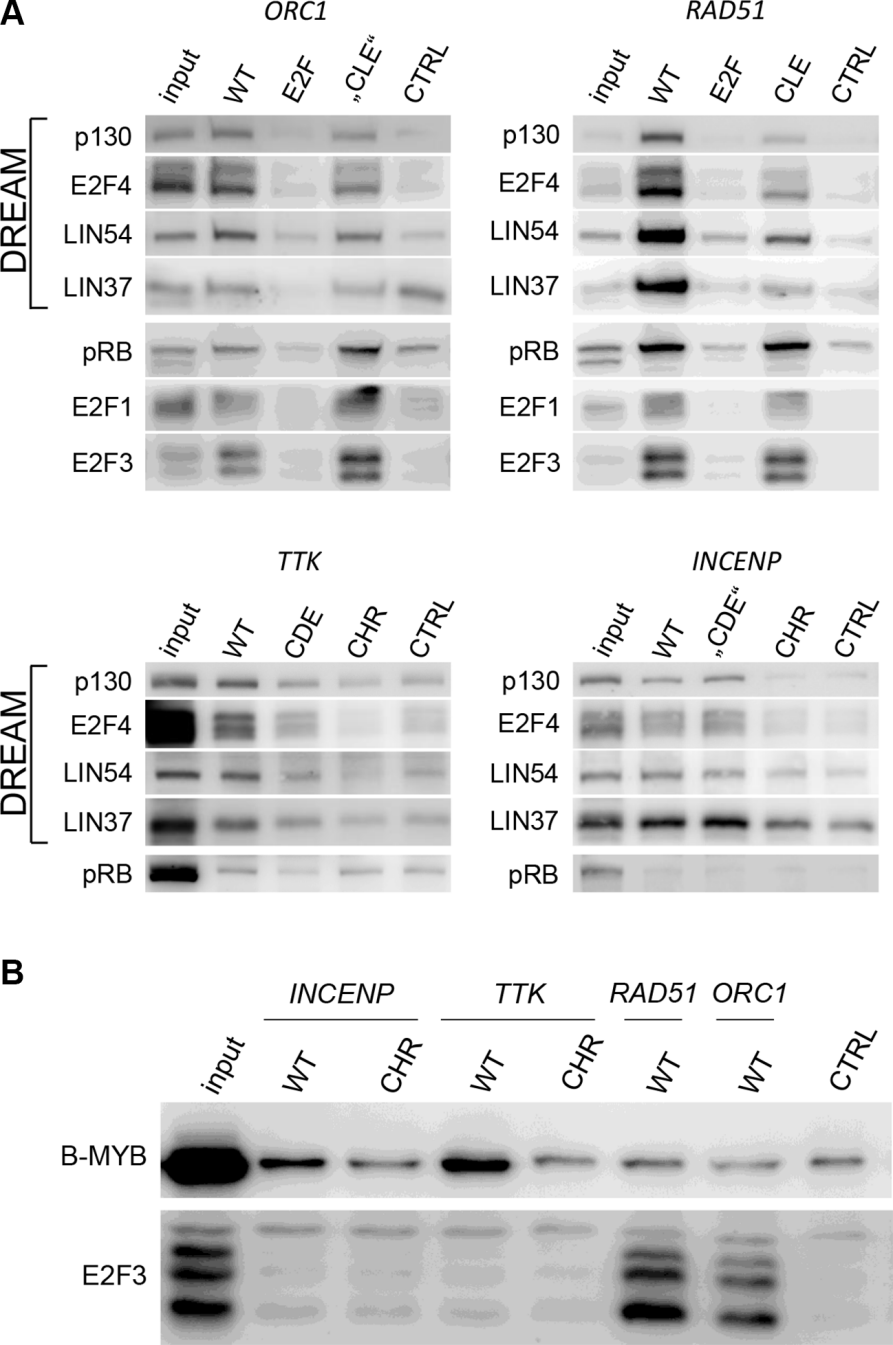
Different promoter elements in early and late cell cycle genes recruit MuvB components, pRB, or activating E2Fs *in vitro* (**A**) DREAM components (p130, E2F4, LIN54, and LIN37) as well as pRB and activating E2Fs (E2F1, E2F3) were purified from nuclear extracts of serum-starved T98G cells by DNA affinity purification and detected by western blot. Binding to wild-type promoter probes (WT) was compared with binding to mutant probes (E2F, CLE, CDE, and CHR). “CDE” and “CLE” represent non-functional elements. Background protein binding was determined with a probe of the *Gapdhs* promoter (CTRL). (**B**) Binding of the MMB components B-MYB and the activating E2F3 was analyzed by DNA affinity purification with nuclear extracts from proliferating T98G cells.

Next, we analyzed DREAM binding to the promoter elements in quiescent cells *in vitro*. To this end, DNA affinity purifications were performed with nuclear extracts of T98G cells arrested in G_0_ by serum starvation. Binding of DREAM components to wild-type or mutant promoter probes was analyzed by western blot (Figure 4A). The DREAM components p130, E2F4, LIN9, and LIN37 bind to the *ORC1* wild-type probe, while mutation of the E2F element reduces binding to background level (Figure 4A). Mutation of the region downstream of the E2F site (TTTTCT) did not influence DREAM binding. In contrast, the CLE in the *RAD51* promoter (TCTGAA) clearly contributes to binding of DREAM. In addition to the observation that DREAM components bind to the *RAD51* probes with a higher affinity than to the *ORC1* probe, mutation of the CLE site results in a reduction of DREAM binding. Importantly, binding is diminished to background level when the E2F site is mutated (Figure 4A). The finding that DREAM binds to E2F sites and E2F/CLE tandem elements was further strengthened by additional DNA affinity purifications performed with probes of the potential E2F/CLE genes *CDC45* and *Mybl2*, as well as with the E2F genes *Atad5* and *Gins1* (Supplementary Figure S1). In all promoters tested, mutation of potential CLE elements resulted in a decrease of DREAM binding to the probes, while mutation of the E2F sites reduced binding to background (Supplementary Figure S1). In contrast, binding of DREAM to the *TTK* promoter clearly depends on the CDE and CHR sites (Figure 4A). Importantly, mutation of the CDE results in reduced binding levels which are still above background, while mutation of the CHR leads to a complete loss of DREAM binding (Figure 4A). This indicates that a CDE supports binding of DREAM to CHR sites but is not sufficient to recruit the complex. In order to test DREAM binding to the *INCENP* promoter, we employed probes mutated in the CHR as well as the region upstream of the CHR where putative CDE sites would be located. The CHR was shown to be required for DREAM recruitment, while mutation of the upstream region does not influence DREAM binding (Figure 4A).

Taken together, we provide evidence that DREAM binds to E2F elements in the promoters of S phase genes and that downstream CLEs can support the interaction, while binding to promoters of late cell cycle genes depends on CHR sites that can be supported by CDEs (Table 1).

### E2F1-3 and activating MuvB complexes are recruited independently of CDE and CLE sites

It is well established that pRB and the activating E2F proteins E2F1 and E2F3 bind promoters through E2F sites. We tested whether these proteins can bind to elements that recruit DREAM. Indeed, binding of pRB, E2F1, and E2F3 to *RAD51*, *CDC45*, *Mybl2, Atad5, and Gins1* promoters depends on the E2F elements. More importantly, CLE sites do not support binding of E2F1/3 or pRB to *RAD51*, *CDC45*, and *Mybl2* promoters (Figure 4A, Supplementary Figure S1). Binding of pRB is also not detected at the CHR or CDE/CHR in the *INCENP* or *TTK* promoters, respectively (Figure 4A).

In proliferating cells, B-MYB as part of MMB binds to the CHR of *INCENP* and *TTK*, but not to the *RAD51* and *ORC1* promoters which lack CHRs. However, E2F3 contacts *RAD51* and *ORC1* promoters with E2F sites but does not bind to CDE/CHR- or CHR-regulated *INCENP* and *TTK* promoters (Figure 4). Taken together, S phase genes are bound by activating E2F proteins through E2F elements, while G_2_/M genes interact with MMB through CHR sites.

### Early cell cycle genes are repressed through the p53-p21-Cyclin/CDK-DREAM-E2F/CLE pathway

**Figure 5:**
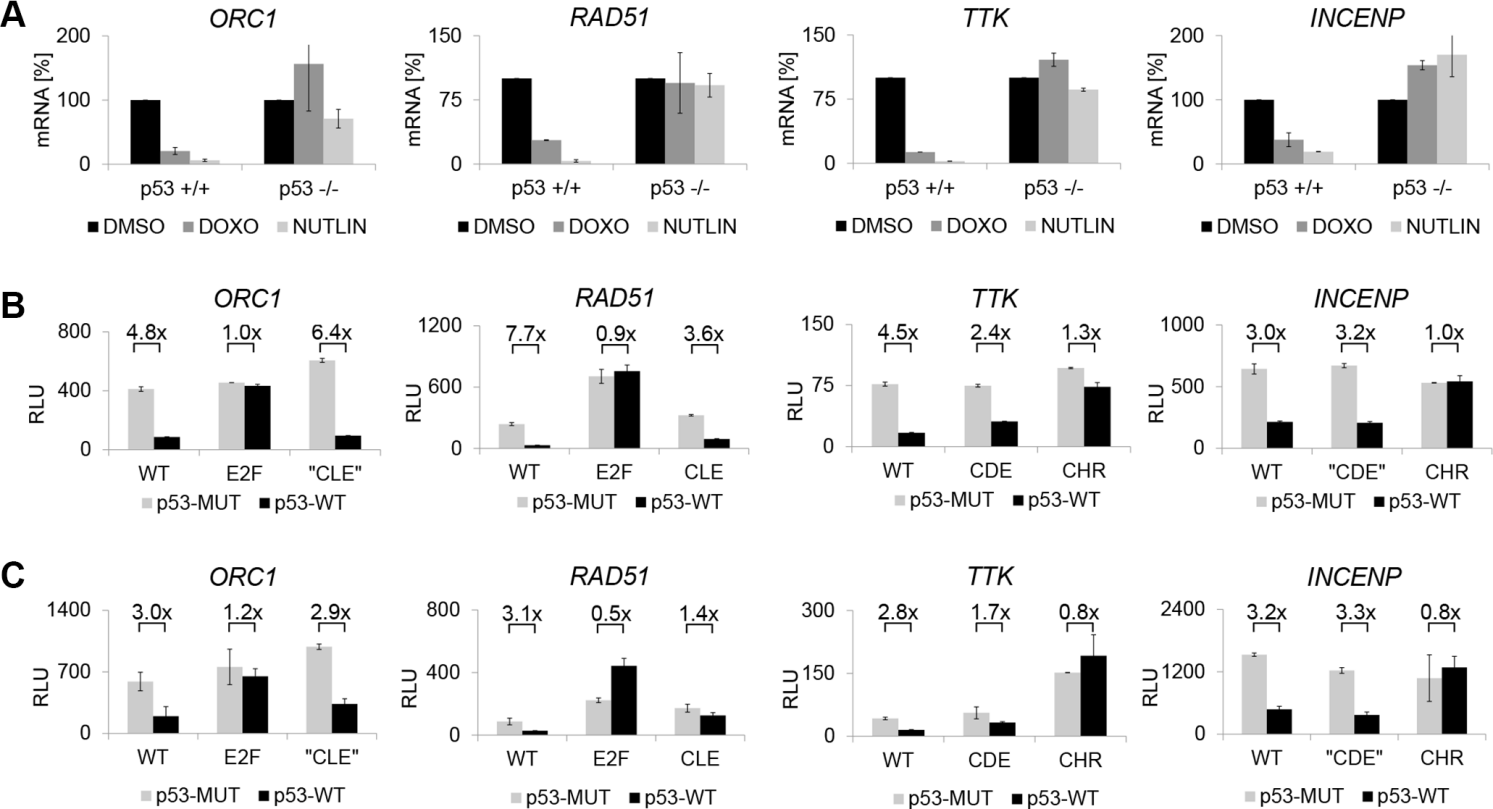
p53-dependent repression of early and late cell cycle genes depends on single or tandem elements and can function in pRB negative cells (**A**) p53-positive (*p53^+/+^*) or negative (*p53^−/−^*) HCT116 cells were treated for 24 h with doxorubicin (DOXO) or Nutlin-3 (NUTLIN) or DMSO. mRNA expression was determined by qPCR. Results were normalized to samples treated with DMSO (100%). (**B**) p53-negative HCT116 cells or (**C**) p53/pRB-negative SaOS-2 cells were transfected with wild-type (WT) and mutant (CDE, CHR, E2F, CLE) promoter reporter constructs together with expression plasmids for p53 wild-type (p53-WT) or a DNA binding-deficient mutant (p53-MUT). 48 h after transfection promoter reporter activities were analyzed by luciferase assays and are given as relative light units (RLU).

It has been established that DREAM binding to CHR and CDE/CHR sites in genes expressed late during the cell cycle is induced by p53 and leads to transcriptional downregulation of the p53 target genes [10, 29]. As DREAM binds to E2F and E2F/CLE sites, the role of these elements in transcriptional repression of early cell cycle genes by p53 was examined. Levels of mRNA from *INCENP*, *TTK*, *RAD51*, *ORC1*, *CDC45*, *Mybl2*, *Atad5*, and *Gins1* decreased after HCT116 cells with wild-type *p53* had been treated with the DNA-damaging drug doxorubicin or the p53-stabilizing agent Nutlin-3 compared to levels in untreated cells. The decrease in mRNA levels was essentially lost when the experiments were performed in HCT116 *p53^−/−^* cells, which indicates that mRNA downregulation requires intact p53 (Figure 5A, Supplementary Figure S1). In order to identify the promoter elements involved in transcriptional repression, HCT116 *p53^−/−^* cells were cotransfected with promoter reporter constructs and plasmids expressing wild-type or a DNA-binding deficient p53. Wild-type promoter constructs with promoters of the late cell cycle genes *TTK* and *INCENP* were repressed by wild-type p53, but the CHR mutants basically failed to respond to p53 expression (Figure 5B). Furthermore, mutation of the CDE in the *TTK* reporter construct resulted in a less pronounced reduction of repression, while mutation of a region upstream of the CHR in the *INCENP* promoter had no influence on this regulation. Importantly, repression of *RAD51*, *ORC1*, *CDC45*, *Mybl2*, *Atad5*, and *Gins1* promoters through p53 required their E2F sites (Figure 5B, Supplementary Figure S1). Interestingly, mutations of the CLE sites in *RAD51*, *CDC45*, and *Mybl2* lead to diminished repression. The observation that CLEs contribute to p53-dependent transcriptional repression, together with the results described above that DREAM binding is supported by CLEs but not binding of E2F1-3/pRB (Figure 4), implies that the response of these genes to p53 is mediated rather by DREAM than by E2F1-3/pRB complexes. To further test this hypothesis, we performed promoter reporter assays in pRB-negative SaOS-2 cells. All promoters examined were still repressed upon p53 overexpression (Figure 4, Supplementary Figure S1). Taken together, the results indicate that the p53-p21-Cyclin/CDK-DREAM-CDE/CHR pathway of p53-dependent gene repression applies also to genes controlled by DREAM binding to E2F or E2F/CLE sites. This novel p53-p21-Cyclin/CDK-DREAM-E2F/CLE pathway functions independent of E2F1-3/pRB complexes.

## DISCUSSION

An important question in cell cycle regulation is how timing of gene expression is controlled. With cyclins, DNA polymerase, kinesins, transcription factors, and kinases as prominent examples, many factors require precise timing of expression for their function in cell cycle regulation. E2F and pRB-related pocket proteins have long been implicated in regulating genes which are not expressed in quiescent cells but become activated at distinct phases during cell cycle progression [30]. With the discovery of the mammalian MuvB-based complexes DREAM, MMB, and FOXM1-MuvB a model with DREAM-dependent repression in G_0_/G_1_ and successive transcriptional activation by E2F1-3/DP, MMB, or FOXM1-MuvB complexes was developed [2–4]. In the initial DREAM descriptions, E2F sites were suggested to mediate DREAM function [3, 4]. Later it became clear that MuvB-derived complexes bind to CHR promoter elements [2, 8, 9, 11]. Furthermore, B-MYB and FOXM1, as other potential DNA binding subunits of MuvB-based complexes, were suggested to contribute not at all or only to a minor extent to DNA binding [8, 16, 17, 31]. These results left open how timing of early and late cell cycle gene expression is correlated with binding of E2F1-3/DP/pRB, DREAM, MMB, or FOXM1-MuvB complexes to E2F, CDE, or CHR promoter sites.

### CDE sites have a low affinity towards E2F proteins and do not bind activating E2F1-3/DP complexes

**Figure 6:**
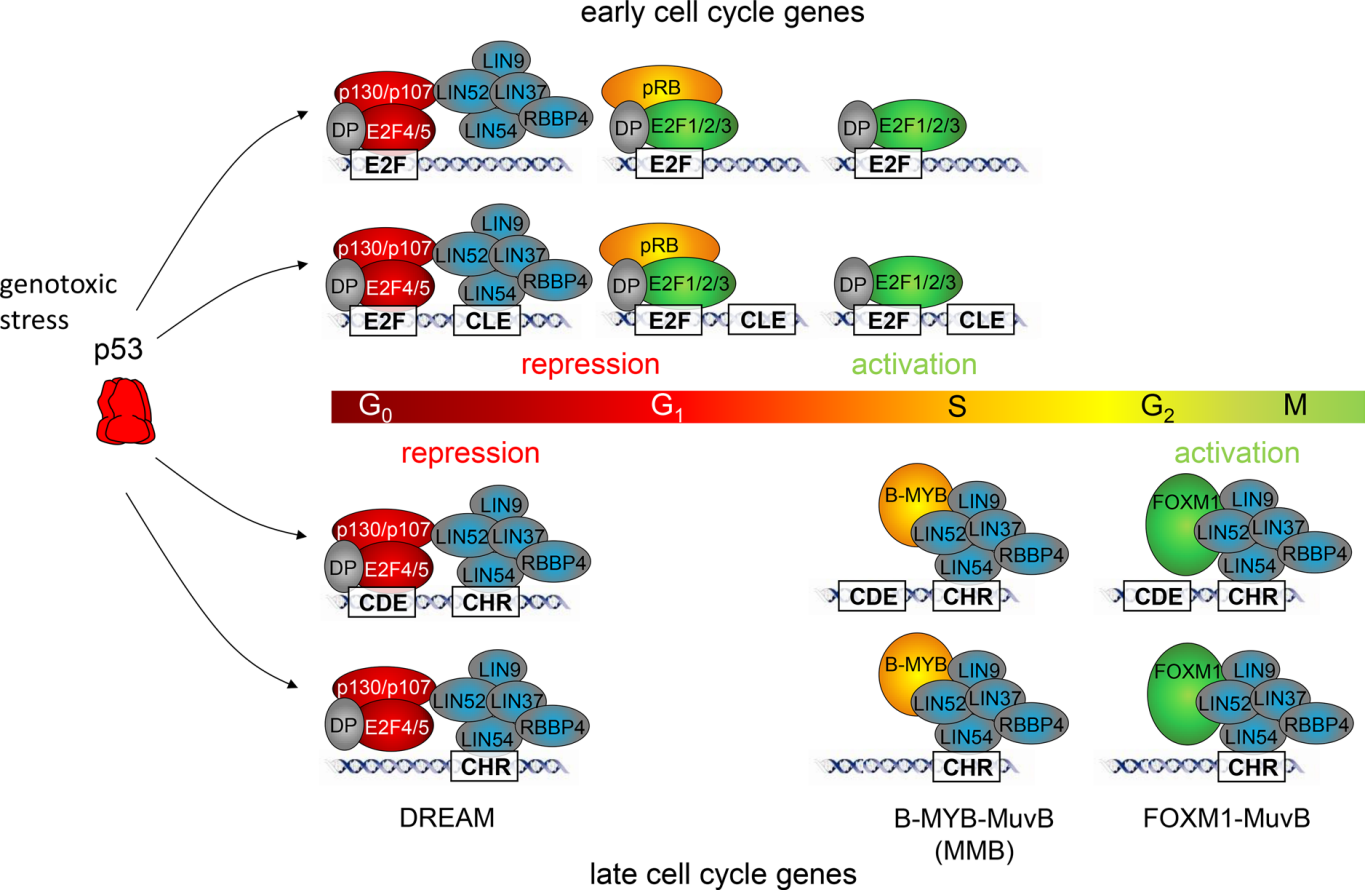
Model for cell cycle-dependent transcriptional regulation of early and late cell cycle genes In G_0_ or upon activation of p53, early and late cell cycle genes are repressed by DREAM. This complex can be recruited to early expressed genes via interactions of E2F4/DP with E2F sites. Interaction of LIN54 with CLE sites can support binding of DREAM. Early cell cycle genes can also be repressed by complexes of E2F1-3, DP, and pRB. CLE sites cannot support binding of pRB complexes. The interaction of LIN54 with CHRs is sufficient to recruit DREAM to late cell cycle gene promoters. CDE sites can support binding through interaction with E2F4/DP. pRB does not bind to CHR or CDE/CHR sites. In proliferating cells, early expressed genes are activated in S phase by E2F1-3/DP complexes interacting with E2F sites. These complexes cannot bind to CHR or CDE/CHR sites of late cell cycle genes. Late cell cycle genes are bound by the MYB-MuvB complex (MMB) in S phase and FOXM1-MuvB which results in maximum expression during G_2_ phase and mitosis.

CDEs are placed with a spacer of four nucleotides upstream of CHR elements [14]. Evidence has accumulated that the function of CDE sites is secondary to that of CHRs in CDE/CHR tandem sites. Often CDEs are functionally dispensable and regulation as well as protein binding solely relies on CHR elements [8, 9, 32] With the discovery that DREAM binds to CDE/CHR sites, it became evident that LIN54 is the MuvB component responsible to contact the CHRs, whereas CDE sites were suggested to bind the E2F4/5/DP components of DREAM [8, 9, 11]. As a typical CDE/CHR-regulated gene expressed during the late cell cycle, we tested *TTK* for protein binding and function of these sites (Figures 1, 2, 3, 4, 5) Cell cycle-dependent transcription depends mostly on the CHR (Figure 3). Interestingly, binding of DREAM components including E2F4 requires primarily the CHR and is only supported by the CDE (Figure 4). These observations suggest that E2F4/5/DP proteins as part of DREAM can only contact CDE sites that are associated with a CHR. In contrast, neither E2F3 nor pRB can bind to the *TTK* promoter (Figure 4). E2F1-3/DP proteins do not associate with MuvB and thus are unable to bind to CDE/CHR promoters with sufficient affinity. The reason for the low affinity of E2F complexes to CDE sites is apparently the variation in the sequences of CDEs from canonical E2F elements [14]. Taken together, our model explains why binding of E2F4/5/DP proteins as components of DREAM is supported by CDE sites to CHR-regulated promoters but no activating E2F1-3/DP complexes are detected on CDE/CHR sites of late cell cycle gene promoters (Figure 6, Table 1).

### The CHR-like element CLE supports DREAM binding to E2F sites in early cell cycle genes

In contrast to CDE sites, E2F elements can bind DREAM without another supporting element as shown here for the examples *ORC1, Gins1*, and *Atad5* (Figure 4A, Supplementary Figure S1). Binding of mammalian DREAM to E2F sites was initially implicated because E2F4/5/DP are DREAM components, but this was not tested in a binding assay [3, 4].

Stimulated by the observation that regulation of several early cell cycle genes through E2F sites is influenced by adjacent promoter regions [33–38], we identified CHR-like elements located four nucleotides downstream of conserved E2F sites. Criteria for CLE sites are that they must differ from the canonical CHR sequence TTTGAA and their affinity to MuvB-based complexes without the assistance of a nearby E2F site must not be sufficient to recruit LIN54 to the promoters. Thus, promoters with CLE sites can be distinguished from CHR-containing genes by binding of B-MYB and FOXM1, as seen for MMB and FOXM1-MuvB binding tested by ChIP-Chip or ChIP-Seq genome-wide studies for many genes [3, 16, 17]. By analyzing protein binding and regulation of *RAD51*, *CDC45*, and *Mybl2* genes, we define CLE sites as a new class of promoter elements (Figures 1, 2, 3, 4, 5, Supplementary Figure S1, Table 1). CLE sites support binding of DREAM to E2F elements. This finding finally provides an explanation why adjacent promoter elements, described here as CLEs, can modulate the transcriptional regulation through E2F sites. Without the E2F site, CLEs are unable to recruit LIN54-based complexes. E2F and E2F/CLE sites are responsible for transcriptional regulation of genes with peak expression during the early cell cycle (Table 1, Figure 6).

### Late cell cycle genes are activated through CHR sites by MMB and FOXM1-MuvB, but not by activating E2F proteins

Activating E2F complexes and pRB cannot bind to CLE or CHR elements (Figure 4). Activation of late cell cycle genes is achieved by MYB-MuvB (MMB) and FOXM1-MuvB binding to CHR sites [9, 16, 17]. When cell progress into S phase, cyclin/cdk complexes hyperphosphorylate p130, leading to a dissociation of the repressing components E2F4/DP/p130 from the MuvB core [15]. After loss of the repressive E2F/p130 proteins in S phase, B-MYB attaches to the MuvB core complex which remains bound to CHR sites (Figure 6). The resulting MMB complex is able to activate expression only after B-MYB is phosphorylated and FOXM1 is recruited. B-MYB is degraded in G_2_ by proteolysis [2, 16].

The transcription factors FOXM1 and B-MYB have been described as DNA-binding proteins [39–41]. However, it was observed that only CHR elements and not canonical forkhead or myb binding sites are enriched in FOXM1-MuvB and MMB target genes and that CHR elements are necessary to recruit the complexes to many late cell cycle genes [8, 17]. The ensuing indirect binding mechanism for FOXM1 is supported by its low affinity to canonical forkhead binding sites [42]. Yet, it was recently shown that the DNA binding domain of FOXM1 is necessary to recruit the protein to DNA and that reduced recruitment of a DNA binding-deficient mutant is not due to inhibition of protein-protein interactions [43]. Thus, the current model reflects that FOXM1 is recruited to CHR sites via the MuvB core complex, but an additional interaction with unspecific non-canonical DNA sequences may be necessary for FOXM1 DNA binding. The notion that CHR sites are the main elements mediating regulation of cell cycle-dependent activity of G_2_/M genes is not affected by this model refinement (Figure 6).

### Transcriptional repression by p53 requires E2F or CHR sites and DREAM binding to these elements is supported by CLE and CDE sites

Another important aspect in regulating cell cycle genes is that the mechanism for transcriptional downregulation in G_0_ overlaps with the mechanism for transcriptional repression induced by p53 [10, 29]. Genotoxic stress causes an accumulation and activation of p53. This transcription factor directly activates the promoter of *p21^CDKN1A^* [44, 45]. p21^CDKN1A^ inhibits the activity of cyclin/cdk complexes that phosphorylate pRB-related p107 and p130 pocket proteins. Hypophosphorylation of p130 causes a shift from activating MMB to repressing DREAM complexes [10, 26]. This shift may be caused by an affinity increase between p130 after hypophosphorylation and LIN52 [15]. The resulting p53-p21-Cyclin/CDK-DREAM-CDE/CHR pathway has been reported on a genome-wide basis as the main mechanism to repress expression of a large number of late cell cycle genes through CHR or CDE/CHR elements [25, 29]. Since early cell cycle genes can bind DREAM or E2F1-3/DP/pRB complexes, it is still not completely understood how these complexes participate in the regulation of these genes. However, increasing evidence suggests that p130/E2F4 is the dominating combination of pocket and E2F proteins in the DNA damage response pathway leading to cell cycle arrest at the G_1_ and G_2_ checkpoints [10, 26, 46–48]. Given that CLE sites in contrast to E2F elements participate in recruiting only DREAM but not E2F1-3/pRB complexes to promoters of early cell cycle genes, discrimination between pRB- and DREAM-mediated repression by comparing activities of wild-type and CLE mutant promoters upon p53 induction should be possible. Indeed, mutation of CLE sites in the promoters of *RAD51*, *CDC45*, and *Mybl2* resulted in a loss of promoter repression which was comparable with the CDE mutant of the *TTK* promoter (Figure 5, Supplementary Figure S1). Importantly, p53-dependent repression in these cells required also the DREAM-binding E2F sites in the promoters of *ORC1, Atad5*, and *Gins1* (Figure 5, Supplementary Figure S1). Moreover, re-expression of p53 resulted in repression of all wild-type promoters in HCT116 *p53^−/−^* cells as well as in *p53^−/−^* and *RB^−/−^* SaOS-2 cells Importantly, these observations suggest that genes expressed early in the cell cycle can be repressed by DREAM also when pRB is absent. Taken together, we show that E2F and E2F/CLE sites binding DREAM are required for p53-dependent transcriptional repression of early cell cycle genes by the p53-p21-Cyclin/CDK-DREAM-E2F/CLE pathway (Figure 6).

### DREAM represses early and late cell cycle genes through binding to E2F, CHR, E2F/CLE, or CDE/CHR sites

In summary, our model suggests that E2F and CHR sites as single elements bind DREAM with strong affinity, causing transcriptional repression (Table 1, Figure 6). DREAM binding can be supported by CLE or CDE sites in the combinations E2F/CLE and CDE/CHR. Genes regulated by E2F sites are expressed with a maximum in S phase. Promoters with CHR elements drive transcription with a maximum in G_2_/M. Transcriptional repression by p53 employs a mechanism of DREAM binding to E2F, E2F/CLE, CDE/CHR, or CHR elements. Activation of early cell cycle genes depends on E2F1-3/DP binding to E2F sites. Late cell cycle genes are activated through binding of LIN54 in the MuvB-based complexes MMB and FOXM1-MuvB to CHR elements. Thus, we present a comprehensive model for differential transcriptional regulation of early and late cell cycle genes.

## MATERIALS AND METHODS

### Cell culture and drug treatment

NIH3T3, HFF, T98G, SaOS-2 cells from DSMZ (Braunschweig, Germany) as well as wild-type and HCT116 *p53^−/−^* cells [49] were cultivated in DMEM supplemented with 10% FCS and penicillin/streptomycin. Cells were synchronized in G_0_ by serum starvation (0% FCS) for 60–72 h. For cell cycle analyses, NIH3T3 and HFF cells were stimulated to re-enter the cell cycle with 20% FCS after the serum-deprivation phase. HCT116 cells were treated with doxorubicin at a final concentration of 0.2 mg/ml for 48 h. The MDM-2 inhibitor Nutlin-3 (Cayman Chemical) was applied at a final concentration of 5 mM for 48 h. DMSO served as solvent control for Nutlin-3 treatment.

### Plasmids

Promoters of *TTK*, *INCENP*, *RAD51*, and *CDC45* were amplified from genomic DNA extracted from human foreskin fibroblasts by standard PCR. Promoters of *Mybl2*, *Gins1* and *Atad5* were amplified from mouse DNA extracted from NIH3T3 cells. DNA fragments were cloned into the pGL4.10 luciferase reporter vector (Promega). Site directed mutagenesis was performed following the QuikChange protocol (Stratagene). Sequences of primers are provided in Supplementary Table S1. The human p53 expression plasmids, pcDNA-p53wt and pcDNA-p53mut (R175H), were described earlier [10].

### DNA affinity purification

DNA affinity purifications were performed as described earlier [9]. Proteins binding to biotinylated promoter probes were purified from with nuclear extracts of serum-starved or proliferating T98G cells and detected by western blot.

### Chromatin immunoprecipitation (ChIP)

ChIPs and quantification of promoter fragments by semi-quantitative RT-PCR were performed as described [8, 9]. Sequences of primers are provided in Supplementary Table S1.

### SDS-PAGE and western blot

SDS-PAGE and western blot were performed following standard protocols as described [50]. The following antibodies were applied for protein detection: E2F4 (C-20), p130 (C-20), E2F1 (C-20), E2F3 (C-18) (Santa Cruz Biotechnology), LIN54 A303-799A (Bethyl Laboratories), LIN9 ab62329 (Abcam), and pRB clone G3-245 (BD Pharmingen). The polyclonal LIN37 antibody (LIN37-T3) was produced by immunizing rabbits with the peptide CRFPHQRRKKRREMDDGLAE followed by affinity purification of specific antibodies (Pineda Antikörper-Service, Berlin, Germany). The monoclonal B-Myb LX015.1 antibody (hybridoma media 1:5) was a kind gift from Roger Watson [51].

### Transfections and luciferase assays

Cell cycle-dependent promoter activities were analyzed by luciferase reporter assays with extracts of transfected serum-starved and re-stimulated NIH3T3 cells as described before [9]. For measuring p53-dependent promoter activity, HCT116 *p5^−/−^* and SaOS-2 cells were plated in 24-well plates (75,000 cells per well) and transfected by GeneJuice (EMD Millipore) with 100ng of promoter reporter plasmids (pGL4.10) along with 50ng of constructs expressing wt or mutant p53.

### Flow cytometry

The DNA content of serum starved and re-stimulated HFF and NIH3T3 cells was analyzed by staining with propidium iodide (PI) followed by flow cytometry as described earlier [9].

### Detection of binding sites in promoter regions

Evolutionary conserved E2F binding sites in core promoters of cell cycle genes with peak expression in S phase or G_1_/S bound by DREAM were identified as described earlier [8]. Potential CLE sites were defined as the six nucleotides starting with a spacer of four nucleotides downstream of an E2F site. In such a tandem element the E2F site can have both orientations.

## SUPPLEMENTARY MATERIALS FIGURES AND TABLES






