# Supplementary material for: Timing of transcription during the cell cycle: Protein complexes binding to E2F, E2F/CLE, CDE/CHR, or CHR promoter elements define early and late cell cycle gene expression
Source: Oncotarget. 2016 Jul 28;8(58):97736–48. doi: 10.18632/oncotarget.10888 (PMC5716687; doi:10.18632/oncotarget.10888)
Supplement: Supplementary file 1 [file oncotarget-08-97736-s001.pdf]

Timing of transcription during the cell cycle: Protein complexes binding to E2F, E2F/CLE, CDE/CHR, or CHR promoter elements define early and late cell cycle gene expression

Supplementary Materials

Supplementary Table S1: Sequences of DNA oligonucleotides used for PCR amplification. see Supplementary_Table_S1

Supplementary Table S2: Identification of potential CHR-like elements (CLEs) by *in silico* analysis. see Supplementary_Table_S2

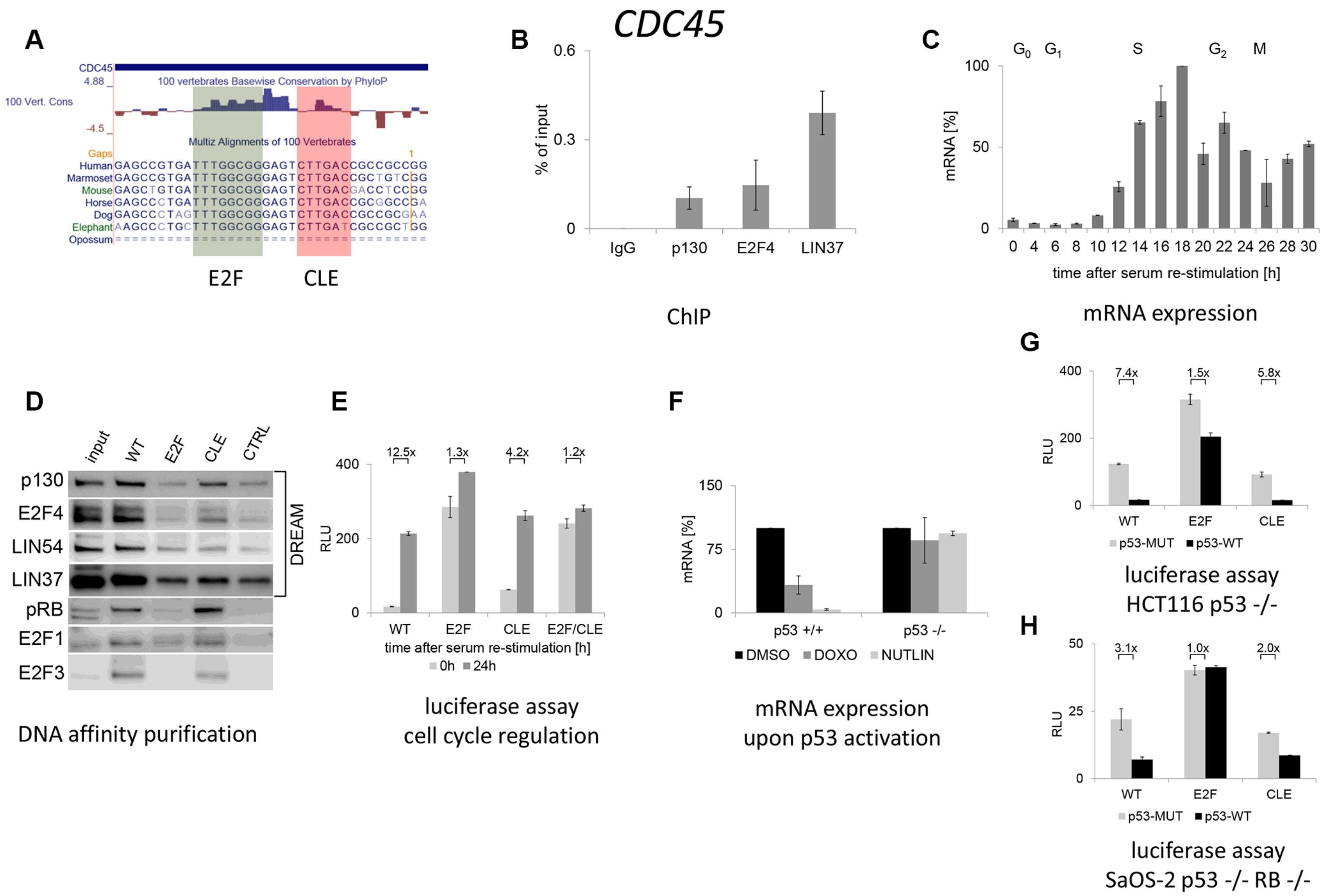

CDC45
A
100 vertebrates Basewise Conservation by PhyloP
100 Vert. Cons
Multiz Alignments of 100 Vertebrates
Human
Marmoset
Mouse
Horse
Dog
Elephant
Opossum
E2F
CLE
B
% of input
IgG
p130
E2F4
LIN37
ChIP
C
mRNA [%]
time after serum re-stimulation [h]
mRNA expression
D
input
WT
E2F
CLE
CTRL
p130
E2F4
LIN54
LIN37
pRB
E2F1
E2F3
DREAM
DNA affinity purification
E
RLU
time after serum re-stimulation [h]
0h
24h
luciferase assay
cell cycle regulation
F
mRNA [%]
p53 +/+
p53 -/-
DMSO
DOXO
NUTLIN
mRNA expression
upon p53 activation
G
RLU
p53-MUT
p53-WT
luciferase assay
HCT116 p53 -/-
H
RLU
p53-MUT
p53-WT
luciferase assay
SaOS-2 p53 -/- RB -/-

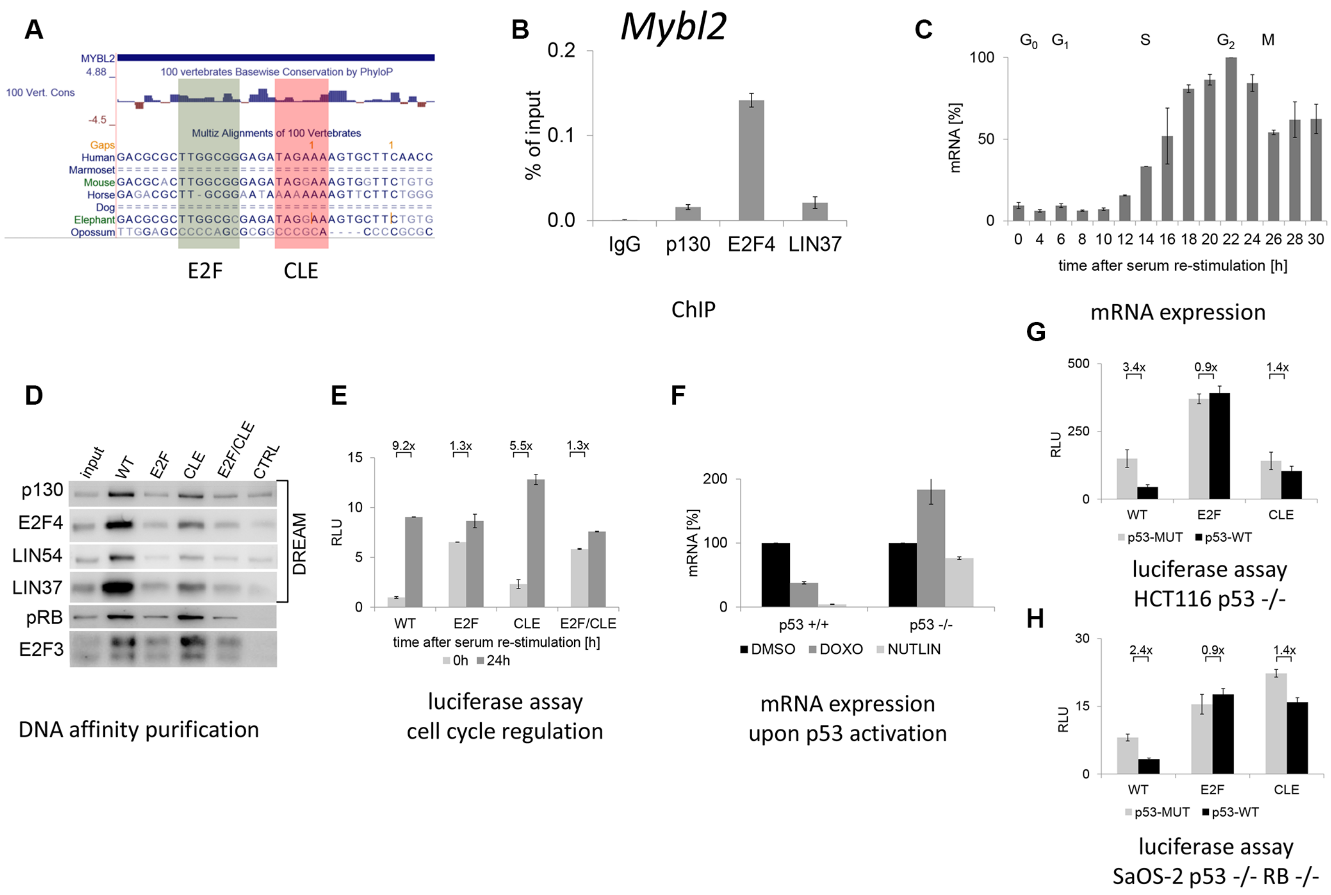

Mybl2
A
100 vertebrates Basewise Conservation by PhyloP
100 Vert. Cons
Multiz Alignments of 100 Vertebrates
Human
Marmoset
Mouse
Horse
Dog
Elephant
Opossum
E2F
CLE
B
% of input
IgG
p130
E2F4
LIN37
ChIP
C
mRNA [%]
time after serum re-stimulation [h]
mRNA expression
D
input
WT
E2F
CLE
E2F/CLE
CTRL
p130
E2F4
LIN54
LIN37
pRB
E2F3
DREAM
DNA affinity purification
E
RLU
time after serum re-stimulation [h]
0h
24h
luciferase assay
cell cycle regulation
F
mRNA [%]
p53 +/+
p53 -/-
DMSO
DOXO
NUTLIN
mRNA expression
upon p53 activation
G
RLU
p53-MUT
p53-WT
luciferase assay
HCT116 p53 -/-
H
RLU
p53-MUT
p53-WT
luciferase assay
SaOS-2 p53 -/- RB -/-

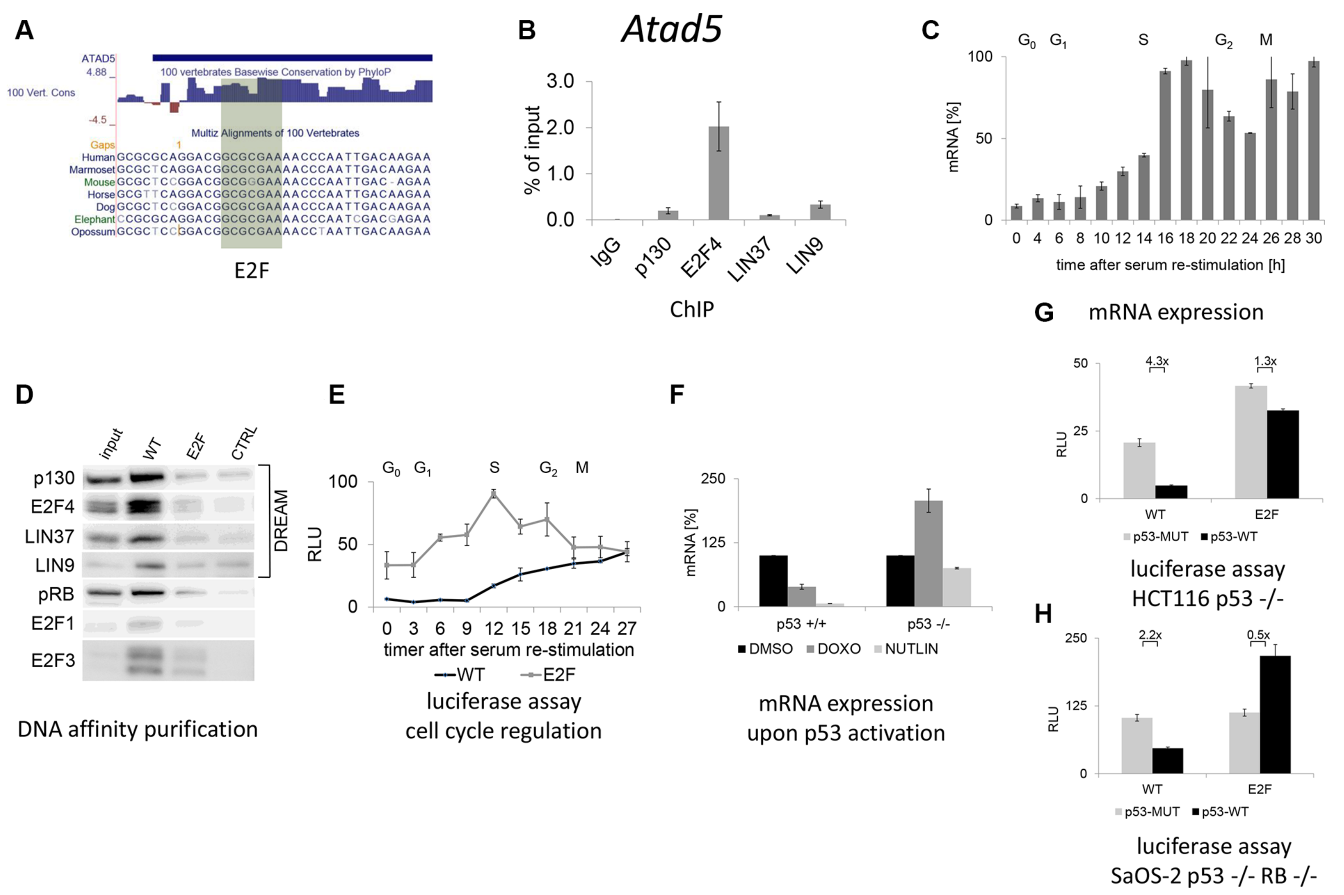
A
ATAD5
4.88
100 vertebrates Basewise Conservation by PhyloP
100 Vert. Cons
-4.5
Multiz Alignments of 100 Vertebrates
Gaps
Human GCGCGCAGGACGGCGCGAAAACCCAATTGACAAGAA
Marmoset GCGCTCAGGACGGCGCGAAAACCCAATTGACAAGAA
Mouse GCGCTCCGGACGGCGCGAAAACCCAATTGAC-AGAA
Horse GCGTTCAGGACGGCGCGAAAACCCAATTGACAAGAA
Dog GCGCTCCGGACGGCGCGAAAACCCAATTGACAAGAA
Elephant CCGCGCAGGACGGCGCGAAAACCCAATCGACGAGAA
Opossum GCGCTCCCGGACGGCGCGAAAACCTAATTGACAAGAA
E2F
B
Atad5
% of input
ChIP
C
mRNA [%]
time after serum re-stimulation [h]
D
input
WT
E2F
CTRL
p130
E2F4
LIN37
LIN9
pRB
E2F1
E2F3
DREAM
DNA affinity purification
E
RLU
timer after serum re-stimulation
WT
E2F
luciferase assay
cell cycle regulation
F
mRNA [%]
p53 +/+
p53 -/-
DMSO
DOXO
NUTLIN
mRNA expression
upon p53 activation
G
mRNA expression
RLU
4.3x
1.3x
WT
E2F
p53-MUT
p53-WT
luciferase assay
HCT116 p53 -/-
H
RLU
2.2x
0.5x
WT
E2F
p53-MUT
p53-WT
luciferase assay
SaOS-2 p53 -/- RB -/-

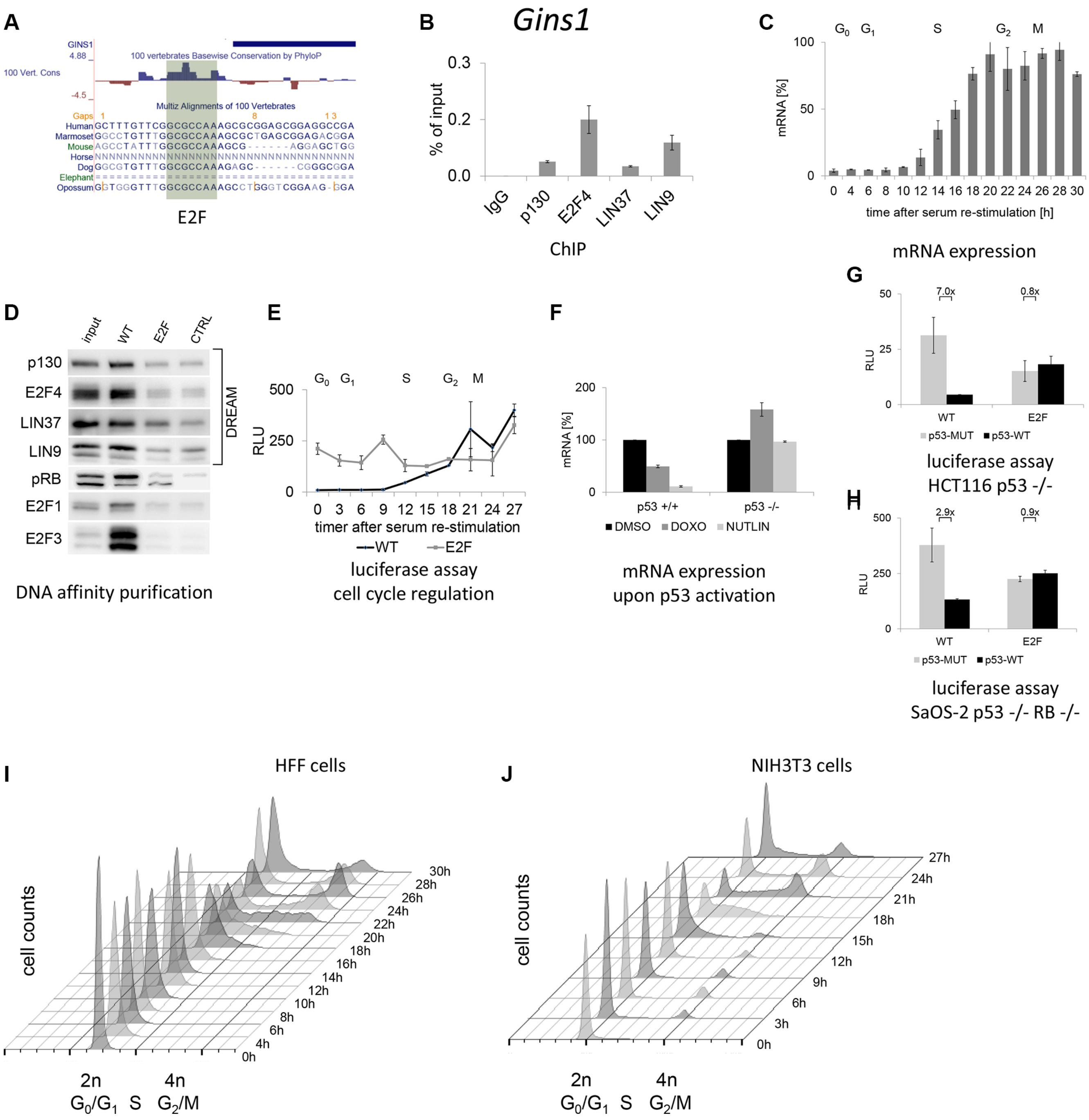

Supplementary Figure S1: Cell cycle- and p53-dependent transcriptional regulation of *CDC45*, *Mybl2*, *Atad5*, and *Gins1* is mediated by E2F or E2F/CLE promoter elements. (**A**) Identification of potential regulatory elements by UCSC genome browser and the 100 vertebrate conservation track analyses. (**B**) Binding of DREAM components in serum-starved T98G cells analyzed by ChIP-qPCR. (**C**) mRNA expression in serum-starved and re-stimulated HFF cells measured by qPCR. (**D**) DNA affinity purification of DREAM components (p130, E2F4, LIN54, LIN37, and LIN9), pRB, E2F1, and E2F3 from nuclear extracts of serum-starved T98G cells with wild-type (WT) and mutant (E2F or CLE) promoter probes. A fragment from the *Gapdhs* promoter (CTRL) served as a negative control. (**E**) Luciferase reporter assays analyzing promoter activities of wild-type (WT) and mutant (E2F, CLE, E2F/CLE) promoters in serum-starved and re-stimulated NIH3T3 cells. (**F**) mRNA expression upon p53 activation by doxorubicin (DOXO) or Nutlin-3 (NUTLIN) in p53-positive ($p53^{+/+}$) or p53-negative ($p53^{-/-}$) cells as measured by qPCR. Promoter activities analyzed by luciferase reporter assays upon expression of wild-type p53 (p53-WT) or a non-DNA-binding p53 mutant (p53-MUT) in p53-negative HCT116 cells (**G**) or p53/pRB-negative SaOS-2 cells (**H**). The DNA content of serum-starved and re-stimulated HFF (**I**) and NIH3T3 (**J**) cells was analyzed by staining with propidium iodide (PI) followed by flow cytometry.
